# Interleukin-13 receptor α2 DNA prime boost vaccine induces tumor immunity in murine tumor models

**DOI:** 10.1186/1479-5876-8-116

**Published:** 2010-11-10

**Authors:** Hideyuki Nakashima, Toshio Fujisawa, Syed R Husain, Raj K Puri

**Affiliations:** 1 Tumor Vaccines and Biotechnology Branch, Division of Cellular and Gene Therapies, Center for Biologics Evaluation and Research, Food and Drug Administration, NIH Building 29B, Room 2NN20, 29 Lincoln Drive MSC 4555, Bethesda, MD, 20892, USA

## Abstract

**Background:**

DNA vaccines represent an attractive approach for cancer treatment by inducing active T cell and B cell immune responses to tumor antigens. Previous studies have shown that interleukin-13 receptor α2 chain (IL-13Rα2), a tumor-associated antigen is a promising target for cancer immunotherapy as high levels of IL-13Rα2 are expressed on a variety of human tumors. To enhance the effectiveness of DNA vaccine, we used extracellular domain of IL-13Rα2 (ECDα2) as a protein-boost against murine tumor models.

**Methods:**

We have developed murine models of tumors naturally expressing IL-13Rα2 (MCA304 sarcoma, 4T1 breast carcinoma) and D5 melanoma tumors transfected with human IL-13Rα2 in syngeneic mice and examined the antitumor activity of DNA vaccine expressing IL-13Rα2 gene with or without ECDα2 protein mixed with CpG and IFA adjuvants as a boost vaccine.

**Results:**

Mice receiving IL-13Rα2 DNA vaccine boosted with ECDα2 protein were superior in exhibiting inhibition of tumor growth, compared to mice receiving DNA vaccine alone, in both prophylactic and therapeutic vaccine settings. In addition, prime-boost vaccination significantly prolonged the survival of mice compared to DNA vaccine alone. Furthermore, ECDα2 booster vaccination increased IFN-γ production and CTL activity against tumor expressing IL-13Rα2. The immunohistochemical analysis showed the infiltration of CD4 and CD8 positive T cells and IFN-γ-induced chemokines (CXCL9 and CXCL10) in regressing tumors of immunized mice. Finally, the prime boost strategy was able to reduce immunosuppressive CD4^+^CD25^+^Foxp3^+ ^regulatory T cells (Tregs) in the spleen and tumor of vaccinated mice.

**Conclusion:**

These results suggest that immunization with IL-13Rα2 DNA vaccine followed by ECDα2 boost mixed with CpG and IFA adjuvants inhibits tumor growth in T cell dependent manner. Thus our results show an enhancement of efficacy of IL-13Rα2 DNA vaccine with ECDα2 protein boost and offers an exciting approach in the development of new DNA vaccine targeting IL-13Rα2 for cancer immunotherapy.

## Background

It is widely known that cancer cells express cell surface molecules such as specific antigens or cytokine receptors [1-3]. These molecules can be used as potential target for immunotherapy, cytotoxin/immunotoxin, or gene therapies. Among these various therapeutic approaches against cancer, tumor vaccines are being developed based on the understanding of the immunologic and genetic property of tumors [1-3]. In contrast to conventional prophylactic vaccines for infectious diseases, therapeutic tumor vaccines currently under development are designed to achieve an active stimulation of the host immune system that induces a non-specific or tumor antigen-specific immune response. These tumor vaccines include whole-cells; cell-lysates; virus and bacteria; peptide or protein; antigen presenting cells such as dendritic cells pulsed with antigen, mRNA or gene modified; tumor cells chemically and/or genetically modified; and tumor antigen peptide- and protein-based vaccines mixed with adjuvant. These vaccines are being tested in animal models and in the clinic [4]. In addition, DNA vaccines are also being tested preclinically and in clinical trials [5]. It has been shown that xenogeneic DNA vaccines not only induce immune response against the "foreign" protein but also generate autoreactive CTLs that recognize the homologous host protein by cross-priming [6,7]. To further enhance the effectiveness of DNA vaccines several strategies are being tested to enhance immune response in patients [8-11].

Among numerous tumor cell surface-associated molecules, the interleukin 13 receptor (IL-13R) α2 chain is overexpressed on certain types of human cancers including glioblastoma, head and neck, kidney, ovarian, breast, and Kaposi's sarcoma [12-20]. This protein is one of the two subunits of the receptor for IL-13, a Th2 cell-derived pleiotropic immune regulatory cytokine [21]. We previously reported that over-expression of the IL-13Rα2 chain in pancreatic and breast cancer cells by stable transfection induces reduced tumorigenicity in athymic nude mice, indicating that the IL-13Rα2 chain is involved in oncogenesis [22]. In addition, we recently demonstrated that IL-13Rα2 is directly involved in cancer invasion and metastasis in human pancreatic cancer models [23].

Because of the selective expression of IL-13Rα2 in several types of tumors but not in normal tissues, we hypothesized that IL-13Rα2 may be a potential target for a cancer vaccine. In this context, we have demonstrated that prophylactic and therapeutic vaccination of immunocompetent mice with D5 melanoma with cDNA vaccine encoding human IL-13Rα2 caused significant antitumor response [24]. Both T cells and B cells played a significant role in immune response against these tumors. Okano *et al*. [25] have identified a CTL epitope in the IL-13Rα2 chain by in vitro stimulation of dendritic cells with synthetic peptides, implying that this receptor chain might serve as a tumor antigen inducing CTL.

In the present study, we evaluated prophylactic and therapeutic effect of the IL-13Rα2 cDNA vaccination in syngeneic animal models of D5 melanoma, MCA304 sarcoma and 4T1 breast cancer cells expressing IL-13Rα2 to prime the immune system. After priming, we boosted animals with extracellular domain of IL-13Rα2 (ECDα2) protein mixed with CpG adjuvant in IFA. This prime-boost strategy resulted in a better tumor response in three tumor models. Tumors from vaccinated mice were infiltrated with CD4^+ ^and CD8^+ ^T cells, resulting in the production of chemokines, which were consistent with the ability of effector cells and molecules to play a role in tumor regression mechanisms. This strategy with IL-13Rα2 cDNA boosted with ECDα2 protein was able to reduce Tregs in spleens and tumors of vaccinated mice.

## Materials and methods

### Cell lines, DNA vaccine, and reagents

D5 melanoma and MCA304 murine sarcoma cell lines were kind gifts from Dr. Bernard A. Fox, Portland, OR, and 4T1 breast carcinoma cell line [26] was purchased from the American Type Culture Collection. Both MCA304 and 4T1 tumors naturally express IL-13Rα2 as determined by RT-PCR analysis (Additional file 1, Figure S1). In contrast, D5 tumor cell line did not express IL-13Rα2 and was stably transfected with human IL-13Rα2 as previously described [24]. In D5α2 model, cDNA encoding the human IL-13Rα2 (termed VRα2) was cloned into the VR1020 [24,27] mammalian expression vector (a kind gift from Vical, Inc., San Diego, CA). For MCA304 and 4T1 model studies, cDNA vaccine encoding the murine IL-13Rα2 was cloned into the VR1012 mammalian expression vector (a kind gift from Vical, Inc., San Diego, CA) using *Kpn*I and *Bgl*II sites, and the sequences of the flanking regions of the junctions were verified by direct sequencing (ABI Prism 310, Applied Biosystems, Foster City, CA). As a negative control, we constructed the irrelevant cDNA plasmid vector, which encoded human IL-2Rγ chain. The resulting constructs were expanded in Escherichia coli and purified using an endotoxin-free EndoFree Giga kit (Qiagen, Inc., Valencia, CA). CpG 1826 [28] was synthesized at FDA/CBER core facility. Incomplete Freund's adjuvant (IFA) was purchased from Sigma, St. Louis, Mo.

### Animals and tumor models

All animal experiments were carried out in accordance with the National Institutes of Health Guidelines for the Care and Use of Laboratory Animals. Four-weeks-old (~20 g in body weight) female C57BL/6 and BALB/c mice were obtained from the Frederick Cancer Center Animal Facilities (National Cancer Institute, Frederick, MD). D5 and MCA304 tumor models were established in C57BL/6 and 4T1 tumor models in BALB/c mice by s.c. injection of 0.5 × 10^6 ^cells in 150 μL of PBS into dorsal flank. Palpable tumors developed within 3 to 4 days. Tumor volumes were determined as previously described [24]. Five to six mice were used for each group.

### Preparation of ECDα2

The ECDα2 protein was expressed and purified in our laboratory [29]. The purity at each step was verified by SDS-PAGE and Western blotting. The purity (>99%) of the final recombinant protein (ECDα2-His6) was verified by SDS-PAGE.

### Immunization with DNA vaccine followed by boost with ECDα2 protein

Animals were immunized i.m. in right (50 μg) and left (50 μg) thighs with VRα2 or control plasmid vector on the indicated days by using a 50 μL Hamilton syringe (total 100 μg/vaccination). Boost vaccination was administrated by i.m. injection of ECDα2 protein (50 μg) or ovalbumin control protein mixed with CpG (50 μg) in IFA (100 μL) in a similar way as DNA vaccination. CpG oligodeoxynucleotides (ODN) was chosen because it acts as immune adjuvant, accelerating and boosting antigen-specific immune responses by 5- to 500-fold [30]. In some cases, IL-2Rγ chain cDNA plasmid was used as an irrelevant negative control.

### IFN-γ assay by ELISA

For IFN-γ release, splenocytes harvested from each group of mice were restimulated with mitomycin C-treated MCA304 or 4T1 tumor cells for 48 h and then the culture supernatant was collected and determined by ELISA kit (e-Bioscience, San Diego, CA) according to the manufacture's instructions.

### CTL assay

Splenocytes from the immunized mice (4 × 10^6 ^per well) were restimulated with 2 × 10^5 ^mitomycin C-treated MCA304 or 4T1 tumor cells in the presence of IL-2 (20 IU/mL) for 1 week in 24-well plates and then used as effector cells for ^51^Cr release assay according to the procedure described in an earlier study [24].

### Immunohistochemistry and immunofluorescence assay

Tumor samples were harvested and fixed with 10% formalin or snap frozen with optimum cutting temperature compound. Sections were then cut at 5 μm and analyzed by immunostaining as previously described [24].

### Flow cytometric analysis

To evaluate CD4^+^CD25^+^Foxp3^+ ^Tregs in splenocytes, cells (1 × 10^6^) were first stained with FITC-conjugated anti-CD4 and PE-conjugated anti-CD25 Abs (e-Bioscience). Cells were then stained using Foxp3 Ab according to the manufacture's instructions (e-Bioscience). A rat IgG2a PE-Cy5 Ab was used as an isotype control. Cells were analyzed using a FACS caliber (Becton Dickinson Immunocytometry Systems).

### Statistical Analysis

The tumor volume in the treatment and control groups was analyzed by ANOVA. Survival curves were generated by Kaplan-Meier method and compared using the log-rank test.

## Results

### Protection from tumor development by prophylactic IL-13Rα2 DNA vaccination boosted with ECDα2 protein in MCA304 sarcoma, 4T1 breast carcinoma, and D5α2 melanoma models

**Figure 1:**
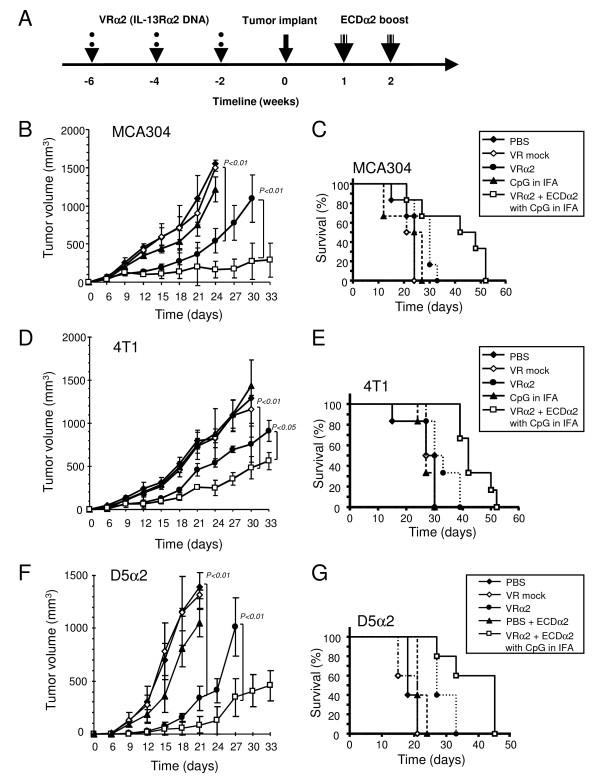
**Prophylactic IL-13Rα2 DNA vaccination and post-tumor challenge boost with ECDα2**. (A) prophylactic DNA vaccination of mice with MCA304, 4T1 and D5α2 tumors. Three IL-13Rα2 DNA vaccine or control vector (100 μg) were injected at two week interval before MCA304 (B and C), 4T1 (D and E), or D5α2 tumor (F and G) challenge in mice (n = 6). The ECDα2 boost vaccinations were injected on week 1 and 2. Tumor volumes were measured by Vernier caliper and Overall Sacrifice Time (OST) was calculated based on the sacrifice of mice when tumors reached to >2 cm. Experiment were repeated twice; *bars*, SD.

We investigated the prophylactic effect of the IL-13Rα2 DNA vaccine followed by boost vaccination with ECDα2 protein mixed with adjuvants on naturally expressing IL-13Rα2 MCA304 sarcoma and 4T1 breast carcinoma tumors in C57BL/6 and BALB/c mice, respectively. We also tested prophylactic vaccination in D5 melanoma tumor transfected with human IL-13Rα2 as D5 did not express IL-13Rα2. The vaccination schedule is shown in Figure 1A. In MCA304 tumor model, ECDα2 boost vaccine showed protection from tumor growth compared to IL-13Rα2 DNA vaccine alone (Figure 1B). The tumor volume in ECDα2 boosted mice at day 27 was significantly smaller (177 mm^3^) than that of the IL-13Rα2 DNA vaccine alone mice (775 mm^3^, P < 0.01). As shown in Figure 1C, overall sacrifice time (OST) of animals (tumor-bearing mice were sacrificed when tumor size reached 2 cm in diameter according to NIH animal guidelines) was 23 days in VR mock vaccinated group, whereas OST of animals was significantly increased to 33 and 51 days in the IL-13Rα2 DNA vaccine alone (P < 0.05) and ECDα2 boosted group (P < 0.01), respectively. Compared with the IL-13Rα2 DNA vaccine alone group, significant prolonged OST was also observed in the ECDα2 boosted group (P < 0.05). Prolonged sacrifice time in the ECDα2 boosted group was almost double compared with the VR mock control group.

Similarly, in 4T1 breast carcinoma and D5α2 melanoma models, IL-13Rα2 DNA vaccine boosted with ECDα2 protein showed significant (P < 0.05) antitumor effect compared to the DNA vaccine alone. (Figure 1D and 1F). OST of animals in 4T1 model was 30 days in control groups, whereas it was significantly (*P *< 0.05) increased to 52 days in the ECDα2 boosted group (Figure 1E). In D5α2 model, OST in prime boost mice (45 days) was significantly longer than control mice (21 days) (Figure 1G). These results demonstrate that ECDα2 boost significantly enhances the efficacy of prophylactic DNA vaccination against the target IL-13Rα2 antigen in MCA304, 4T1 and D5α2 tumor models.

### Prophylactic IL-13Rα2 DNA and boost vaccinations induce CTL activity and IFN-γ release in MCA304 and 4T1 tumor models

**Figure 2:**
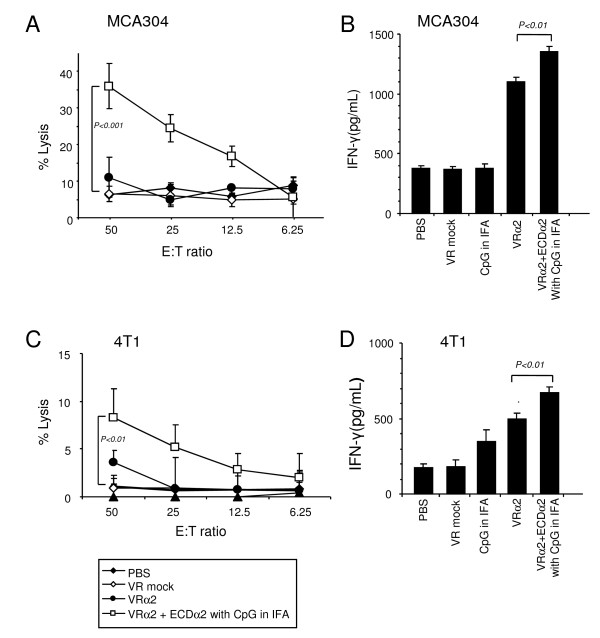
**Measurement of CTL activity and IFN-γ release in mice vaccinated with prophylactic IL-13Rα2 DNA and boosted with ECDα2**. Splenocytes restimulated with MCA304 (A) or 4T1 (C) tumor cells for 1 week in culture medium containing IL-2 (20 IU/mL) were used as effector cells. MCA304 or 4T1 target cells labeled with ^51^Cr for 2 hours, washed thrice, and then plated into 96 well plates with effector cells. Specific lysis was calculated as described in materials and methods after 4 hours of culture. Culture supernatants of splenocytes restimulated with mitomycin C-treated MCA304 (B) or 4T1 (D) tumor cells for 48 hours and were assessed by ELISA for murine IFN-γ production. Experiments were repeated twice; *bars*, SD.

To assess whether tumor protection caused by prophylactic vaccination was mediated by CD8^+ ^T cells, we performed CTL assays and measured IFN-γ release in two tumor models. Splenocytes from the ECDα2 boosted mice caused specific lysis of MCA304 target cells; 38% lysis at an E/T ratio of 50:1, significantly (P < 0.001) higher than that of control group (7%) (Figure 2A). However, the % lysis of tumor cells in VRα2 group was not much different from the control group. Furthermore, IL-13Rα2 DNA vaccine alone group released more than 1,100 pg/mL of IFN-γ. However, the ECDα2 boosted groups released 1,400 pg/mL of IFN-γ. In contrast, splenocytes from the control mice showed low levels INF-γ release of ~400 pg/mL (Figure 2B). Similar results were observed with the 4T1 tumor model for CTL activity and IFN-γ release (Figure 2C and 2D). These results indicate that IL-13Rα2 DNA prime and ECDα2 boost vaccination induces specific CTL activity and IFN-γ release in both MCA304 and 4T1 tumor models. Vaccination with IL-13Rα2 DNA alone also induced IFN-γ release but it did not show a difference in cytotoxicity compared to control group most likely due to sensitivity of the assay.

### Therapeutic IL-13Rα2 DNA and boost vaccination inhibited established MCA304, 4T1, and D5α2 tumor growth

**Figure 3:**
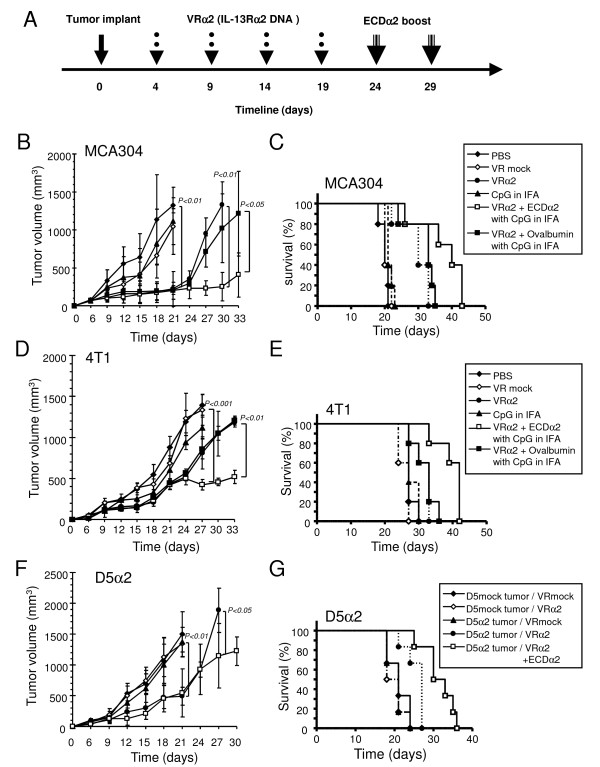
**Therapeutic IL-13Rα2 DNA and boost vaccination inhibited established MCA304, 4T1, and D5α2 tumor growth**. (A) Therapeutic vaccination schedule in tumor bearing mice. Palpable tumors were established in 3 to 5 days. Mice (n = 6 per group) were vaccinated as shown in Figure 3A. The ECDα2 boosted mice showed significant inhibition of tumor growth compared to IL-13Rα2 DNA vaccine alone in MCA304 (B), 4T1 (D) and D5α2 (F) tumor models. Kaplan-Meier survival curves of MCA304 (C), 4T1 (E) and D5α2 (G) tumor models were plotted. Ovalbumin, an irrelevant protein boost was used as a negative control. CpG in IFA served as negative control for ECDα2 protein. Experiments were repeated twice; *bars*, SD.

Having identified the efficacy of the IL-13Rα2 DNA and ECDα2 boost vaccination in the prevention of MCA304, 4T1, and D5α2 tumor growth, we tested efficacy of this vaccine in mice with established tumors to simulate a clinical situation. Treatment schedule is shown in Figure 3A. Mice with MCA304 tumors showed inhibition of tumor growth when vaccinated with IL-13Rα2 DNA vaccine alone (Figure 3B). Further boost with ECDα2 protein continued to show inhibition of tumor growth during the treatment schedule. On day 30, the tumor volume of MCA304 tumors in mice receiving the ECDα2 boost protein (252 mm^3^) was significantly smaller than that of mice receiving the IL-13Rα2 DNA vaccine alone (1334 mm^3^) (P < 0.01). To confirm IL-13Rα2 specific immune response, we used ovalbumin as an irrelevant protein for boost vaccination. Ovalbumin boost did not inhibit tumor growth as ECDα2 did (Figure 3B). This tumor growth pattern was the same as the IL-13Rα2 DNA vaccine alone, indicating that the boost with ECDα2 generated IL-13Rα2 specific immune response. OST of the mice was 21 days in PBS treated group, whereas it was significantly increased to 32 and 43 days in the IL-13Rα2 DNA vaccine alone group (P < 0.01) and ECDα2 boosted group (P < 0.01), respectively (Figure 3C). Compared with DNA vaccine alone, significant prolonged survival time was observed in ECDα2 boosted mice (P < 0.05). It is interesting to note that ECDα2 boost prolonged survival time to more than double (43 days) compared with the PBS group (21 days). In addition, irrelevant cDNA plasmid vector encoding human IL-2Rγ_c _showed no inhibition on tumor growth which was similar to the VR mock vaccinated group (data not shown).

Similar results were observed in 4T1 breast cancer and D5α2 melanoma models. Mice receiving ECDα2 boost protein showed significant antitumor effect as evident by inhibition of tumor growth and increase in OST compared to the mice receiving DNA vaccine alone in both cancer models (Figure 3D-G). These results indicate that therapeutic murine IL-13Rα2 DNA prime and ECDα2 boost vaccination could be effective in reducing tumor burdens in MCA304, 4T1, and D5α2 tumor bearing mice, not only in the prophylactic but the therapeutic setting too.

### Therapeutic vaccination induces CTL activity against established MCA304 and 4T1 tumor cells and antibody production against IL-13Rα2

**Figure 4:**
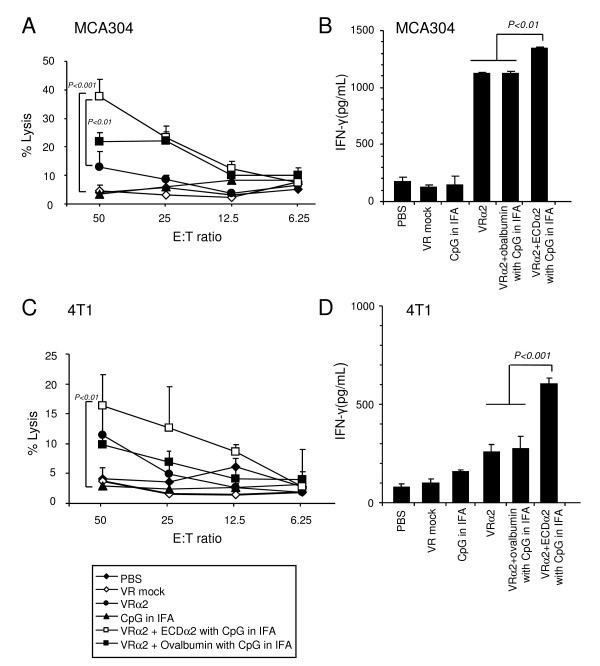
**Induction of CTL activity and IFN-γ production by therapeutic IL-13Rα2 DNA vaccination and boost in established MCA304 and 4T1 tumor models**. CTL-mediated specific lysis for MCA304 (A) and 4T1 (C) tumor is measured as described in Figure 2. Splenocytes harvested from mice (on day 33) were prepared for measurement of murine IFN-γ production in MCA304 (B) and 4T1 (D) tumor group. Experiments were repeated twice; *bars*, SD.

To assess whether the antitumor effect of the IL-13Rα2 DNA and boost vaccination were associated with induction of CTL against two tumor MCA304 and 4T1 models, IFN-γ production and CTL activity were examined. For CTL, splenocytes from MCA304 tumor-bearing mice were harvested on day 33 and restimulated with mitomycin-c treated MCA304 tumor cells for one week. The percent lysis of the ECDα2 boosted group was ~40% at an E/T ratio of 50:1 which was significantly (P < 0.001) higher than that of the IL-13Rα2 DNA vaccine alone group (12%) (Figure 4A). In contrast, splenocytes from the control mice showed much lower levels of lysis of MCA304 target cells (5%).

The splenocytes from IL-13Rα2 DNA vaccine alone group released over 1,100 pg/mL of IFN-γ (Figure 4B). Furthermore, the ECDα2 boosted mice released 1,300 pg/mL of IFN-γ. In contrast, splenocytes from the control mice released low levels of IFN-γ (200 pg/mL). Similar results were observed with the 4T1 breast cancer model (Figure 4C and 4D). These results suggest that the treatment of MCA304 and 4T1 tumor-bearing mice with murine IL-13Rα2 DNA and the ECDα2 boost vaccination induced or amplified a specific CTL response and IFN-γ release against sarcoma and breast tumors in the established tumor setting.

We have previously demonstrated that splenocytes from C57BL/6 mice challenged with mouse melanoma (D5α2) when vaccinated with IL-13Rα2 DNA, mediated a significant lysis of target cells (38% lysis at E/T 50:1) [24]. However, in current study in sarcoma model (MCA304), a significantly lower lysis was observed (13% lysis at E/T 50:1) although this lysis was enhanced by boosting mice with ECDα2 protein (38% lysis at E/T 50:1). Similar results were observed for IFN-γ release in both tumor models. The splenocyte culture supernatants from mice treated with IL-13Rα2 DNA vaccine in D5α2 model released 1281 to 1541 pg/mL of IFN-γ [24]. In MCA304 model, it released 1100 pg/mL of IFN-γ in the vaccinated mice (Figure 4B). In 4T1 tumor model, lowest cytotoxicity of target cells and lowest amount of IFN-γ release was observed (Figure 4C and 4D). These observations suggest that mice with melanoma tumors with human IL-13Rα2 (D5α2) elicit more robust immune response compared to naturally expressing murine MCA304 and 4T1 tumors. This difference may be due to xeno antigen in D5α2 tumors or differential expression of IL-13Rα2 between tumors.

We also examined the effect of prime and boost vaccination on IL-13Rα2 specific antibody production. Serum samples collected from mice with MCA304 tumor on days 33 in Figure 3B showed antibody response against IL-13Rα2 as quantified by ELISA (See additional file 2, Figure S2). The antibody against IL-13Rα2 in mice receiving IL-13Rα2 DNA and ECDα2 boost vaccination was dramatically higher than IL-13Rα2 DNA and ovalbumin vaccinated mice.

### Infiltration of CD4^+ ^and CD8^+ ^T cells in tumors of immunized mice

**Figure 5:**
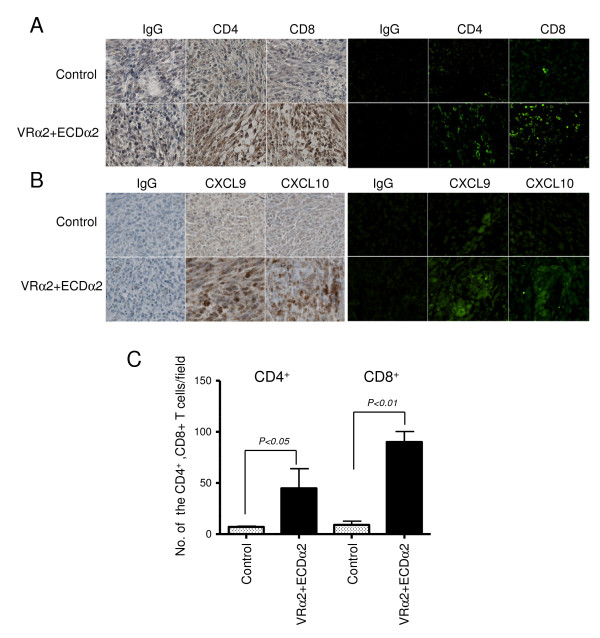
**Detection of CD4^+ ^and CD8^+ ^T-cells and chemokines in regressing tumors of vaccinated mice**. The MCA304 tumor samples in mice receiving PBS, the IL-13Rα2 DNA prime and ECDα2 boost vaccination were collected on days 33 from the experiment shown in Figure 3B and the immunohistochemistry and immunofluorescense microscopic analyses were done using antibodies specific for CD4 and CD8 (A and C) or CXCL9 and CXCL10 (B). IgG2 antibodies were used for isotype control (B). Three sections from each tumor samples were evaluated. The dark brown stained cells in Panel A indicate CD4^+ ^and CD8^+ ^T-cells. Magnification X20. Representative data from experiments done twice with a total 6 mice per group.

To examine whether CD4^+ ^and CD8^+ ^T cells were infiltrated in tumors that produced chemokines is consistent with the ability of effector cells and molecules to play a role in tumor regression mechanisms, we assessed the infiltration of CD4^+ ^and CD8^+ ^T cells, as well as expression of IFN-γ related chemokines (CXCL9 and CXCL10) in established MCA304 tumors of mice receiving the IL-13Rα2 DNA and boost vaccination. The tumor samples were collected on day 33 from the mice of Figure 3B and then immunohistochemistry and immunofluorescense microscopic analysis were done using specific antibodies. The higher density of CD4^+ ^and CD8^+ ^T cells were identified in tumor samples of boost vaccinated mice compared to control tumors (Figure 5A). The number of CD4^+ ^cells (results were average of three view fields) was 7 in control tumor and 44 in ECDα2 boosted mice (P < 0.05). The number of CD8^+ ^cells was 9 in control tumor and 90 in ECDα2 boosted mice (P < 0.01) (Figure 5C).

Tumor samples were also stained with anti-MIG/CXCL9 or anti-IP10/CXCL10 antibodies (Figure 5B). These chemokines were selected because they have been shown to be involved in the CTL-induced tumor regression [31-33]. Tumor samples of IL-13Rα2 DNA and ECDα2 boost vaccine-treated mice collected were positive for CXCL9, whereas control tumor samples were negative for this chemokine. However, CXCL10 was more strongly positive in tumor samples of vaccinated mice. These results suggest that therapeutic IL-13Rα2 DNA prime and ECDα2 boost vaccine-induced regression of MCA304 tumor involved infiltration of CD4^+ ^and CD8^+ ^T cells and the production of certain chemokines in tumors.

### Therapeutic prime-boost vaccination decreased the expression of regulatory T cells

**Figure 6:**
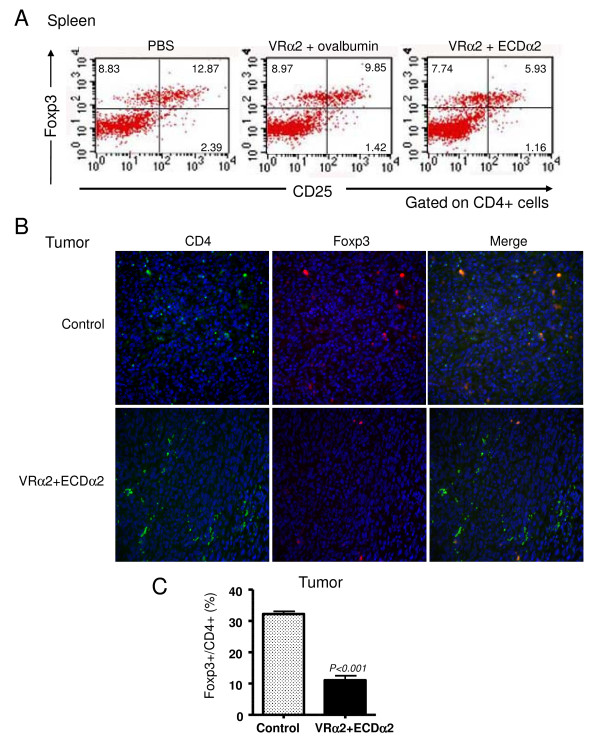
**Therapeutic prime-boost vaccination decreased the expression of regulatory T cells**. (A) FACS analysis of regulatory T cells in splenocytes of vaccinated mice with MCA304 tumors. Splenocytes collected from Figure 3B on day 33 were stained with anti-CD4, CD25, Foxp3 and rat IgG2a (isotype control) antibodies and analyzed by FACS. Representative expression of CD25 and Foxp3 gated on CD4^+ ^cells is shown. Results are representative of three independent experiments. (B) The MCA304 tumor samples from Figure 5 were stained with antibodies specific for anti-CD4 and anti-Foxp3 and counterstained with 4',6-diamidino-2-phenylindole. The number of positive cells were counted and plotted in C. Tumor sections were quantitated for ≥2 mice per group with a minimum of two sections per tumor for each staining condition, with five fields per section used for counting.

We also investigated the effect of therapeutic vaccination boosted with ECDα2 on number of CD4^+^CD25^+^Foxp3^+ ^Tregs in the spleens and tumors of mice from Figure 3B. Tregs were measured by flow cytometry in CD4^+ ^lymphocytes from splenocytes of these mice. The number of Tregs in the PBS and VRα2 plus ovalbumin control groups were 12.9% and 9.9%, respectively (Figure 6A). However, the number of Tregs in the ECDα2 boosted group was 5.9%. To further confirm that the number of Tregs infiltrated into tumor was also associated with the population of Tregs in spleen, immunohistochemistry was performed on same tumor samples obtained from Figure 3B. Interestingly, tumor samples from ECDα2 boosted mice shown smaller number of the ratio of Foxp3^+^/CD4^+ ^(12%) compared with that of control mice (30%, P < 0.001) (Figure 6B and 6C). These results indicate that in addition to the generation of IL-13Rα2-specific immune response, prime-boost vaccination strategy decreased immunosuppressive Tregs in spleen and tumor to further enhance the efficacy of the vaccine.

## Discussion

IL-13Rα2 is overexpressed on certain types of human tumor tissues [15-22]. We now provide evidence that IL-13Rα2 is highly expressed in a variety of murine tumor cell lines (Additional file 1, Figure S1). Although the significance of expression of IL-13Rα2 in cancer is not completely clear, our previous studies indicate that IL-13Rα2 could be linked to oncogenesis and metastasis and may provide a potential target for immunotherapy [23,24]. We have extended our prior studies and hypothesized that immunization with a DNA vaccine encoding murine IL-13Rα2, boosted with ECDα2 protein, may work more effectively in syngeneic murine tumor models. We studied three murine tumor models, MCA304 sarcoma, 4T1 breast cancer and D5α2 melanoma. Our results indicate that this strategy can produce significant anti tumor effect in these tumor models using both prophylactic and therapeutic vaccinations.

To our knowledge, this is the first report of using ECDα2 protein in DNA prime-protein boost strategy to enhance the efficacy of DNA vaccine. It has been hypothesized that the use of two versions of the same immunogen may activate different subsets of immune cells. It has been shown that DNA immunization is more effective in inducing CD4^+ ^T-cell responses and priming antigen-specific B cells, whereas protein immunization is more effective in stimulating the proliferation of memory B cells into antibody-secreting plasma cells [34]. In our study, the IL-13Rα2 DNA prime and ECDα2 protein boost activated CD4^+ ^and CD8^+ ^T cell responses and enhanced antibody response against IL-13Rα2. These T and B cell responses induced by prime-boost strategy correlated with tumor responses causing reduced tumor burden and significantly prolonging mice survival, compared with the IL-13Rα2 DNA vaccine alone.

The involvement of systemic immunity in mediating antitumor effects was confirmed by (a) induction of tumor-specific CTL response, (b) IFN-γ secretion by splenocytes, and (c) infiltration of CD4^+ ^and CD8+ T cells in tumors that secreted tumor reactive chemokines. Splenocytes collected from control mice produced minimal level of IFN-γ when they were restimulated with MCA304 or 4T1 tumor cells. These splenocytes also mediated low level of lysis of each target cells as determined by CTL assays. However, each tumor cell-restimulated splenocytes collected from mice receiving the IL-13Rα2 DNA vaccine boosted with ECDα2 produced substantial levels of IFN-γ in the culture supernatant and was capable of mediating specific lysis of each target cells. In contrast, ovalbumin, an irrelevant protein boost did not further induce CTL response, and thus, we conclude that antitumor effects mediated by this vaccination strategy were murine IL-13Rα2 DNA specific.

It is reported that 4T1 breast tumor is highly metastatic and weakly immunogenic [35,36]. Huang *et al. *showed that parental 4T1 tumor cells expressing only MHC class I molecules are poorly immunogenic, and immunizations of mice bearing 4T1 breast tumor with the irradiated 4T1 cells alone failed to induce the protective antitumor immune responses [37]. It has also been reported that 4T1 cell line elaborates a variety of immune suppressive molecules including PGE-2, TGF-β and other factors [38]. These molecules are the reasons that 4T1 tumor is poorly immunogenic to induce antitumor response. Other examples of poorly immunogenic tumors have been described in the literature. Kjaergaard *et al*. explained several reasons for poor immunogenicity of B16/D5 mouse melanoma tumors in response to the therapeutic effects of OX-40R mAb [39]. These authors proposed that it is possible that B16/D5 tumor cells either lack molecules that can serve sufficiently as tumor antigens recognized by T cells or are deficient in the processing, transportation or presentation of such molecules by APCs. It may also be true for the poorly immunogenic 4T1 tumors of eliciting lower T cell responses. Indeed, in our study, overall CTL activity and IFN-γ production in 4T1 tumor model were lower compared to MCA304 tumor model. However, the IL-13Rα2 DNA and ECDα2 boost vaccination could be effective in reducing tumor burdens and induce or amplify a specific CTL response and IFN-γ release against 4T1 tumors compared with the IL-13Rα2 DNA vaccine alone.

It is noteworthy that the IFN-γ-related chemokines CXCL9 and CXCL10 were expressed in tumors derived from mice receiving the IL-13Rα2 DNA vaccine boosted with ECDα2. CXCL9 is known to function as a potent chemoattractant for tumor infiltrating lymphocytes [31]. In addition, the CXCL10 displays antitumor properties based on the attraction of monocytes and T lymphocytes [40]. Our results suggest that chemokines are most likely produced by infiltrating immune cells causing antitumor effect because these chemokines act as potent T cell chemoattracants and angiogenesis inhibitors through their interaction with CXCL3 [31-33].

DNA vaccination and IL-13Rα2 protein boost produced anti-IL-13Rα2 antibody in the serum of mice. This antibody may be directly cytotoxic to tumor cells or mediate growth inhibitory signal to target cells after ligating with IL-13Rα2 antigen. We are currently examining the role of antibody in tumor rejection in the current prime boost model. We have previously reported that vaccination of human IL-13Rα2 cDNA alone in D5α2 model generated antibodies, which were modestly cytotoxic to D5α2 tumor cells in vitro [24].

Interestingly, mice vaccinated with therapeutic IL-13Rα2 cDNA vaccine and boosted with ECDα2 protein showed lower percentage of Tregs in the spleen and tumor compared to the PBS control in the MCA304 tumor model. This is an interesting finding as Tregs play a prominent role in the inhibition of anti-tumor immunity. It is possible that the inhibitory effects of IL-13Rα2 DNA boosted with ECDα2 protein vaccination on Tregs expansion will play a potentially important role in clinical efficacy during the treatment of immunocompromised patients, such as those with cancer. The enhanced expansion of Tregs has been reported in a number of solid and hematological cancers [41-44]. Our results suggest that IL-13Rα2 cDNA boosted with ECDα2 protein vaccination may enhance anti-tumor-immunity by inhibiting the suppressive effects of Tregs.

We did not observe any visible toxicity in mice vaccinated with IL-13Rα2 DNA alone or in combination with ECDα2 protein. No visual changes in animal behavior, mobility, and body weight were observed after vaccination. Histopathological analyses of vital organs (liver, kidney, lung, spleen, heart, and brain) manifested no abnormalities in vaccinated group compared to no treatment group (data not shown). For future clinical trials, we recommend to carefully observe patients by physical exams, serum chemistry, complete blood count and any sign of autoimmunity.

Many immunotherapy approaches, including therapeutic tumor vaccines targeting specific tumor antigens are being developed [1-3]. Our current results may be extrapolated to the clinical setting, and it is possible that both CD4^+ ^and CD8^+ ^T cells will be induced against IL-13Rα2 antigen by the DNA vaccine regimen as observed in this animal study. Although the prime-boost vaccine mediated regression of established tumor, complete responses were not observed in any of three tumor models tested. It is possible that the heterogeneous expression of IL-13Rα2 in tumors is responsible for this effect. Alternatively, a most effective dose of vaccine or schedule of vaccination was not optimized. A more immunogenic vector such as vaccinia virus and/or other virus expressing the IL-13Rα2 and/or an IL-13Rα2 peptide vaccine mixed with adjuvants may be needed to generate robust immune responses. These types of preclinical studies will be needed to translate our observations to the clinic for the treatment of patients with cancer.

## Conclusion

Our results suggest that immunization with IL-13Rα2 DNA vaccine followed by ECDα2 boost mixed with CpG and IFA adjuvants mediates significant antitumor effects in T cell dependent manner. Thus, IL-13Rα2 can serve as a potent tumor antigen that can recruit immune responses against IL-13Rα2 expressing solid tumors.

## Abbreviations

IL-13: interleukin-13; IL-13Rα2: interleukin-13 receptor α2; ECDα2: extracellular domain alpha 2; SC: subcutaneous; CTL: cytotoxic T lymphocytes; IFN-γ: Interferon-gamma; OST: overall sacrifice time.

## Competing interests

The authors declare that they have no competing interests.

## Authors' contributions

Conceived and designed the experiments: SRH, RKP. Performed the experiments: HN, TF. Analyzed the data: HN, TF, SRH. Wrote the paper: HN, SRH, RKP.

All authors have read and approved the final manuscript.

## Supplementary Material

Additional file 1 **Figure S1. Differential expression of IL-13Rα2 chain in murine tumor cell lines**. The expression of IL-13Rα2 in murine tumor cell lines was examined by analyzing expression of mRNA with RT-PCR. Murine tumor cell lines were tested including three sarcoma cell lines, MCA106, MCA304 and MCA310; two melanoma cell lines, B16 and D5; one glioma cell line, GL261; and one breast cancer cell line, 4T1). High levels of mRNA expression of IL-13Rα2 in three sarcoma cell lines and 4T1 breast cancer cell line was observed. On the other hand, B16, D5 melanoma and GL261 glioma cell lines showed low or undetectable level of IL-13Rα2 mRNA. The primers for
mIL-13Rα2 used were: 5'-CGC-ATT-TGT-CAG-AGC-ATT-GT-3' (forward) and 5'-CCA-AGC-CCT-CAT-ACC-AGA-AA-3' (reverse). Click here for file

Additional file 2 **Figure S2. IL-13Rα2 DNA boosted with ECDα2 vaccination generated autoantibodies in serum**. To measure the antibody levels in mice, blood serum samples were periodically collected on days 33 from the experiment shown in Figure 3B. Autoantibody against IL-13Rα2 was quantified by ELISA using with standard techniques. Briefly, 96-well plates were coated with a mouse IL-13Rα2 Fc recombinant protein (10 μg/ml; R&D Systems) for capture overnight at 4°C. Serum samples (100 μl per well) diluted 1:1000 in blocking solution were assayed in duplicate and incubated with the plate at room temperature for 1 h. Wells were washed and then incubated with biotinylated anti-mouse IL-13Rα2 Ab (0.5 μg/ml; R&D Systems) for another 1 h. This was followed by streptavidin-HRP conjugated and substrate solution (R&D systems) at room temperature for 20 min each. Absorbance was read at 450 nm. These data demonstrate that generation of antibody against IL-13Rα2 by the mice receiving IL-13Rα2 DNA and ECDα2 boost vaccination was dramatically increased compared with IL-13Rα2 DNA and ovalbumin vaccinated mice. Click here for file
