# Supplementary figures and images for: Interleukin-13 receptor α2 DNA prime boost vaccine induces tumor immunity in murine tumor models
Source: J Transl Med. 2010 Nov 10;8:116. doi: 10.1186/1479-5876-8-116 (PMC2993653; doi:10.1186/1479-5876-8-116)

## Slide 1
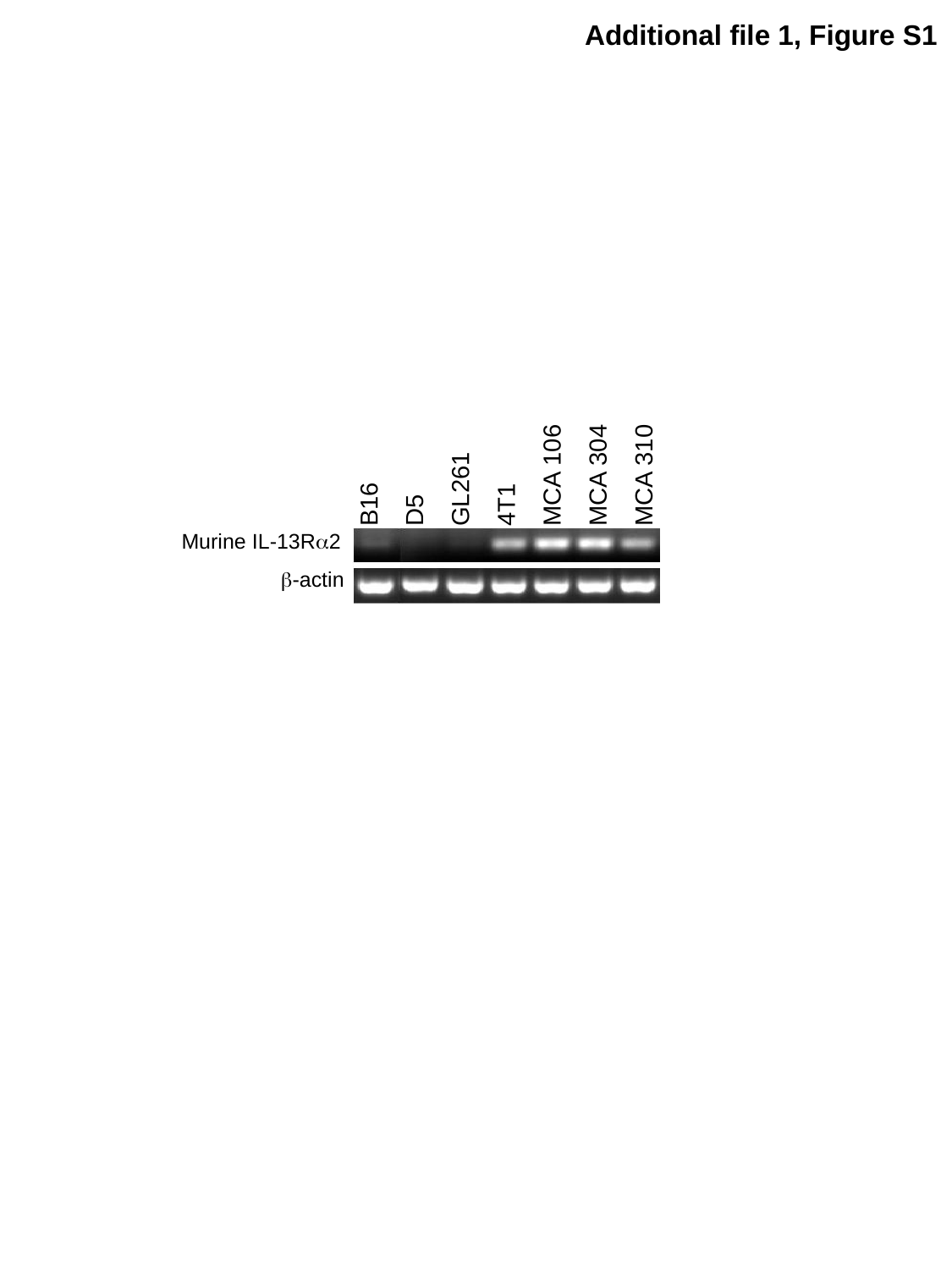

Additional file 1, Figure S1
MCA 304
MCA 310
MCA 106
GL261
B16
4T1
D5
Murine IL-13Rα2
β-actin

Supplement: Additional file 1 — Figure S1. Differential expression of IL-13Rα2 chain in murine tumor cell lines. The expression of IL-13Rα2 in murine tumor cell lines was examined by analyzing expression of mRNA with RT-PCR. Murine tumor cell lines were tested including three sarcoma cell lines, MCA106, MCA304 and MCA310; two melanoma cell lines, B16 and D5; one glioma cell line, GL261; and one breast cancer cell line, 4T1). High levels of mRNA expression of IL-13Rα2 in three sarcoma cell lines and 4T1 breast cancer cell line was observed. On the other hand, B16, D5 melanoma and GL261 glioma cell lines showed low or undetectable level of IL-13Rα2 mRNA. The primers for mIL-13Rα2 used were: 5'-CGC-ATT-TGT-CAG-AGC-ATT-GT-3' (forward) and 5'-CCA-AGC-CCT-CAT-ACC-AGA-AA-3' (reverse). [file 1479-5876-8-116-S1.PPT]

## Slide 1
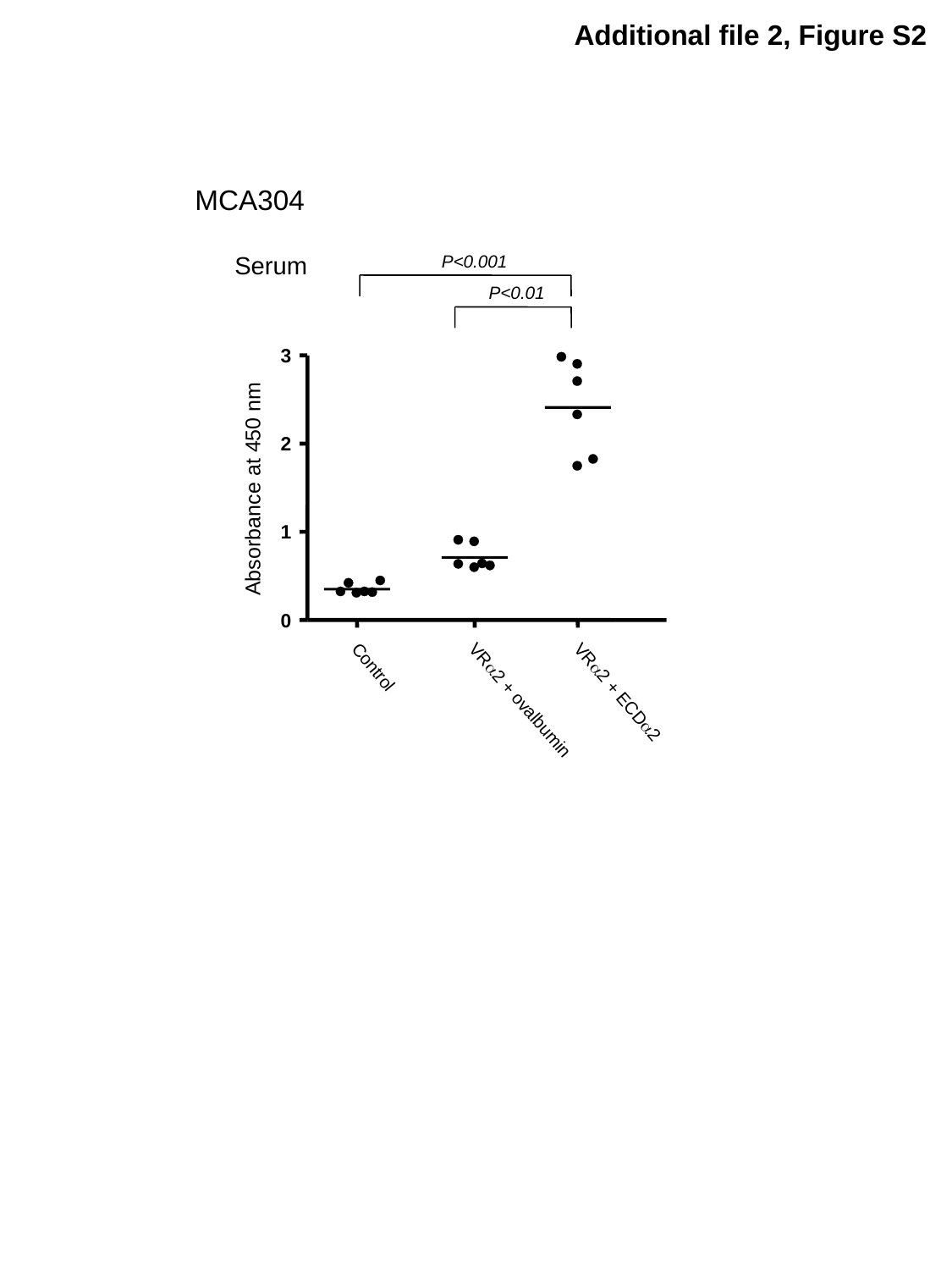

Additional file 2, Figure S2
MCA304
Serum
P<0.001
P<0.01
3
2
Absorbance at 450 nm
1
0
Control
VRα2 + ECDα2
VRα2 + ovalbumin

Supplement: Additional file 2 — Figure S2. IL-13Rα2 DNA boosted with ECDα2 vaccination generated autoantibodies in serum. To measure the antibody levels in mice, blood serum samples were periodically collected on days 33 from the experiment shown in Figure 3B. Autoantibody against IL-13Rα2 was quantified by ELISA using with standard techniques. Briefly, 96-well plates were coated with a mouse IL-13Rα2 Fc recombinant protein (10 μg/ml; R&D Systems) for capture overnight at 4°C. Serum samples (100 μl per well) diluted 1:1000 in blocking solution were assayed in duplicate and incubated with the plate at room temperature for 1 h. Wells were washed and then incubated with biotinylated anti-mouse IL-13Rα2 Ab (0.5 μg/ml; R&D Systems) for another 1 h. This was followed by streptavidin-HRP conjugated and substrate solution (R&D systems) at room temperature for 20 min each. Absorbance was read at 450 nm. These data demonstrate that generation of antibody against IL-13Rα2 by the mice receiving IL-13Rα2 DNA and ECDα2 boost vaccination was dramatically increased compared with IL-13Rα2 DNA and ovalbumin vaccinated mice. [file 1479-5876-8-116-S2.PPT]
